# Allergic manifestations and cutaneous histamine responses in patients with McCune Albright syndrome

**DOI:** 10.1186/1939-4551-6-9

**Published:** 2013-05-01

**Authors:** Jill D Jacobson, Angela L Turpin, Scott A Sands

**Affiliations:** 1 Section of Endocrinology, Children’s Mercy Hospital, University of Missouri-Kansas City, School of Medicine, 2401 Gillham Road, Kansas, MO 64108, USA

**Keywords:** McCune Albright syndrome, Histamine responsiveness, Wheal and flare, Atopy, Anaphylaxis

## Abstract

**Background:**

McCune Albright syndrome (MAS) is a rare disorder characterized by precocious puberty, café-au-lait spots, and fibrous dysplasia. Its cause is an activating mutation in the GNAS gene, encoding a subunit of the stimulatory G protein, G_s_alpha (G_s_α). The action of any mediator that signals via G_s_α and cyclic AMP can be up regulated in MAS. We had observed gastritis, gastroesophageal reflux, and anaphylaxis in McCune Albright patients.

**Objective:**

As histamine is known to signal via histamine 1 (H1) and histamine 2 (H2) receptors, which couple with stimulatory G proteins, we attempted to mechanistically link histamine responsiveness to the activating GNAS mutation. We hypothesized that responsiveness to histamine skin testing would differ between MAS patients and healthy controls.

**Patients and methods:**

After obtaining informed consent, we performed a systematic review of histamine responsiveness and allergic manifestations in 11 MAS patients and 11 sex-matched, Tanner-stage matched controls. We performed skin prick testing, quantifying the orthogonal diameters of wheals and erythema. We also quantitated G protein mRNA expression.

**Results:**

The peak wheal and flare responses to histamine were significantly higher in MAS patients compared to controls.

**Conclusions:**

This study suggests that MAS patients may be at risk for exaggerated histamine responsiveness compared to unaffected controls.

## Introduction

McCune-Albright syndrome (MAS) is a rare, intriguing, genetic disorder characterized by excessive activity of multiple hormones and other mediators that signal through cAMP. The original description by Fuller Albright included the triad of precocious puberty, café-au-lait skin spots, and polyostotic fibrous dysplasia [1]. The causative mutation, identified in 1991, is a missense mutation in the GNAS gene, which leads to constitutive activation of the G_s_α protein [2]. Mutations in the GNAS gene occur after conception, and the individual survives with mosaicism. As a result of this mosaicism, individuals with MAS display a wide spectrum of clinical severity and a wide range of endocrine manifestations. Purely endocrine features described in MAS include non-autoimmune hyperthyroidism, acromegaly, Cushing syndrome, hyperprolactinemia, and hyperparathyroidism. Bony problems include polyostotic fibrous dysplasia [3-7]. Several non-endocrine features more recently attributed to the molecular defect include platelet abnormalities, hepatobiliary disease, cardiac hypertrophy, and sudden or premature death [2,8-11]. The ongoing recognition of additional G protein-mediated signaling abnormalities in MAS may eventually lead to an increased number of supportive diagnostic criteria to aid in accurately diagnosing MAS.

We noted that a large number of our patients with MAS displayed a wide variety of atopic manifestations and hypersensitivities. Reactions ranged from asthma, drug and food sensitivities, contact dermatitis, and even anaphylaxis. In fact, over a period of three years, five MAS patients experienced anaphylaxis. We speculated that these observations were linked to the constitutive activation of the stimulatory G protein, G_s_α.

Our initial observations of severe reactions to foods and inhalant allergies suggested classic IgE-mediated problems. The IgE receptor cross-linking process is mediated by tyrosine kinases, and is therefore not G protein-mediated. However, we note that G protein-coupled receptors (GPCRs) and cyclic AMP modulate numerous responses in neurological, immune, muscular, and vascular systems that contribute to allergic processes, asthma, and atopy. Classic examples of stimulatory G protein-coupled ligands that play crucial roles in atopy are histamine and leukotrienes [12,13]. Stimulatory G protein-coupled ligands such as acetylcholine and epinephrine play crucial roles in bronchoconstriction [14,15]. Examples of stimulatory G protein-coupled ligands that play crucial roles in vasomotor function are substance P and catecholamines [16,17].

We noted that two of our MAS patients suffered from severe gastritis and gastroesophageal reflux. Such findings are suggestive of H2 receptor activation. We speculated that some of our observations of hypersensitivities in MAS patients might be linked to exaggerated histaminic signaling augmented by the constitutive activity of G_s_α protein. However, of the four major histamine receptors, H1 through H4, only the H2 receptor is classically described as coupling to G_s_α and cAMP [18-20]. Activation of this H2 receptor is traditionally not associated with allergy, but rather with gastric histaminic activity. In contrast, the histamine 1 (H1) receptor, which is traditionally associated with allergy and urticaria, is coupled to a separate stimulatory G protein of the G_q_α pathway [20]. H3 and H4 receptors exert actions via inhibitory G proteins, which generally mediate cAMP inhibition [21,22].

Many lines of evidence suggest “cross talk” between G_s_α pathway (which traditionally signals via cAMP) and G_q/11_α pathway (which traditionally signals via inositol phosphates) in the signaling of guanine nucleotide binding (G) protein coupled receptors (GPCRs), including histamine receptors [13,20,23-27]. Constitutive co-expression of a 5- hydroxytryptamine receptor with the G_q/11_ α coupled histamine 1 receptor results in increased agonist-independent signaling, which can be augmented by agonist activation of various other G protein coupled receptors, including muscarinic and adenosine receptors [28].

Previous studies have demonstrated that skin reactivity to histamine testing varies with the menstrual cycle in both healthy and atopic women, with heightened reactivity seen in midcyle, corresponding to peak estrogen and gonadotropin stimulation [29,30]. We have previously reported that exposure to either estrogen or to gonadotropin-releasing hormone (GnRH) stimulates G_q/11_α mRNA expression in immune cells in mice [31,32]. Because patients with MAS exhibit post-receptor activation of GnRH, LH, and FSH receptors, we speculated that other G protein signaling pathways might be up regulated in MAS.

Herein, we hypothesized that patients with MAS would demonstrate evidence of increased H1 and/or H2 activity. We measured wheal and flare skin responses to histamine compared to Tanner Stage matched, sex-matched, unaffected controls. We also sought evidence for increased allergic phenomena in MAS patients by systematically reviewing their allergic histories. We quantified expression of G_s_α mRNA and G_q_α mRNA and protein in peripheral blood mononuclear cells (PBMCs). We also sought a correlation between histamine responsiveness and activation of the hypothalamic-pituitary-gonadal axis, as measured by Tanner staging.

## Patients and methods

### Study design

This is a single-center, prospective, nonrandomized study comparing patients with the clinical diagnosis of MAS to Tanner stage-matched, sex-matched healthy controls. The study and all procedures were approved by the Institutional Review Board and performed in compliance with the World Medical Association Declaration of Helsinki regarding the ethical conduct of research. All participants were evaluated over a 3 year period beginning in August of 2004. Two separate consent forms were utilized for G protein measurement, Tanner staging, allergic histories, IgE measurements, and skin testing. Histories of allergic histories and medical therapies for allergies were obtained retrospectively. Menstrual histories were taken in female participants. Pubertal development was assessed by Tanner stage of pubic hair and breast development (girls) and testicular volume (boys) [33].

### MAS participants

We identified 12 MAS patients who were regularly followed in our endocrinology clinic. The diagnosis was diagnosed based on clinical criteria. Patients were required to exhibit at least two major features of MAS (precocious puberty, polyostotic fibrous dysplasia, and café-au-lait spots). All patients underwent endocrine evaluations including thyroid function testing at least annually. Only one female patient with MAS exhibited regular menstrual cycles. After obtaining informed consent, we obtained information about adverse drug and food reactions retrospectively from the medical record charts. One out of the 12 patients was unable to participate in the skin testing for geographic reasons. This patient had a history of anaphylaxis necessitating epinephrine administration after receiving radiographic contrast. The clinical characteristics of the 11 participating patients with MAS are shown in Table 1.

**Table 1:** Clinical manifestations of patients with MAS

**Pt #**	**Age (Yr)**	**Sex**	**Tanner stage**	**Polyostotic fibrous dysplasia**	**Characteristic café-au- lait spots**	**Gonadotropin independent precocious puberty**	**Non autoimmune thyroid disease**	**# MAS features**	**Max flare (mm)**	**Max wheal (mm)**	**Manifestations of atopy/hypersensitivity**
1	17	M	5	Yes	Yes	Yes	Yes	4	107	21	anaphylaxis (latex), drug allergies (morphine, penicillin), asthma H. Pylori negative ulcer Other sensitivities (topical benzocaine)
2	15	F	5	No	Yes	Yes	No	2	156	18	anaphylaxis (strawberries), drug allergies (ibuprofen, tylenol), multiple food allergies, chronic gastritis
3	12	F	3	Yes	Yes	Yes	No	3	105	11	anaphylaxis (cashews), atopic dermatititis,drug allergy (erythromycin), Other sensitivities (nickel, surgical tape)
4	13	F	3	Yes	Yes	Yes	No	3	98	7	anaphylaxis (penicillin)
5	13	F	3	Yes	Yes	Yes	No	3	100	17	positive skin testing (ragweed, dust mites), asthma, atopic dermatitis, chronic urticaria
6	10	F	1	Yes	Yes	No	Yes	3	70	12	multiple drug allergies (methimazole, propylthiouracil)
7	8	F	3	Yes	Yes	Yes	No	3	112	10	drug allergy (letrozole) Other sensitivities (gluten, surgical tape), chronic urticaria
8	17	F	5	Yes	Yes	Yes	No	3	98	14	asthma, latex allergy
9	7	F	2	No	Yes	Yes	No	2	75	18	atopic dermatitis, seasonal rhinitis, chronic urticaria
10	7	F	2	No	Yes	Yes	No	2	92	14	asthma, seasonal rhinitis
11	16	M	5	Yes	Yes	No	No	2	135	17	none

### Control participants

Healthy control children with no increased risk for allergies were recruited by an institutional electronic advertisement. An allergy questionnaire was administered to all patients and controls. Allergic manifestations in controls were similar to that seen in the general population. One out of eleven (9%) controls reported seasonal rhinitis. No other allergic phenomena were reported in control participants.

Controls were Tanner Stage-matched to MAS patients. Because MAS patients exhibited precocious puberty, we matched patients and controls by Tanner Stage rather than by age. In order to match by age, we would have had to use controls with precocious puberty in order to age match.

Eleven Tanner stage-matched, sex-matched control patients underwent skin testing. Two of these control participants had undergone Tanner staging for clinical care purposes. The Tanner stage(s) of these individuals was therefore not recorded in the research record. Two female control participants had already achieved menstrual regularity. A second set of 11 sex-matched, Tanner stage-matched control participants did not undergo skin testing, but were recruited specifically for G protein measurement, in order to increase the sample size of controls.

### Power analysis

Pilot data in mice demonstrated that early exposure to estradiol leads to a three-fold increase in expression of mRNA for stimulatory G proteins in various tissues. As MAS patients exhibit early activation of the HPG axis, we speculated that similar differences might be seen in humans. A power analysis revealed that an n of 8 would yield an 80% power to detect similar differences in G protein mRNA expression. Thus our patient population of 11 would offer adequate power to detect similar differences in G protein mRNA expression.

### Skin testing

**Figure 1:**
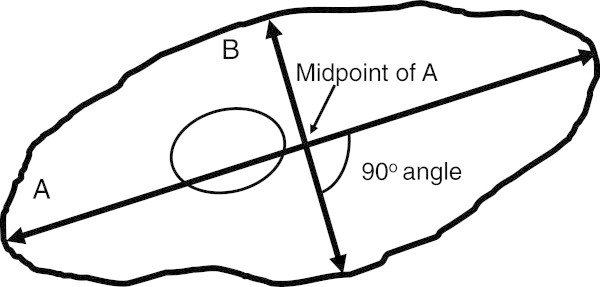
Diagram depicting the method of measurement of the sums of wheal and flare (Σ of flare) responses to histamine skin testing.

If patients had received antihistamines or glucocorticoids in the two weeks prior to IRB consent, they were excluded and consented when they were no longer requiring these medications. Blood was drawn for IgE measurement. Participants then underwent standard allergy testing using the commercially available Greer Skin testing system as a prick test device (Greer Labs, Lenoir, N.C.). Histamine (Hollister-Stier Laboratories, Spokane, WA) was used at a concentration of 6 mg/ml. Codeine (20 mg/ml; Hospira, Inc., Lake Forest, IL) was used as a control whose actions are mediated by IgE independent histamine release [34]. It signals via both stimulatory and inhibitory G proteins [35]. Normal saline was also used as a negative control. Tests were spaced at least 3 cm apart to avoid difficulty with interpretation. Two standard sites were used for skin testing for all participants, the back and the arm. When cutaneous café-au-lait spots were present on standard sites on the back or arm (n = 4), the solutions were also applied to the contralateral unaffected skin site. Data from the unaffected skin sites only were used in comparisons between MAS patients and controls. All testing was performed by a single trained allergy nurse. Wheal and flare responses were measured at 7 and 20 minutes after the skin tests were administered. The wheal and flare responses were outlined in ink, and the image transferred to hypoallergenic transparent tape to create a permanent record. Tape was placed in a permanent record book for measurement. Thus, measurement was performed with blindness to the skin pigmentation. The longest diameter of erythema was measured. A line was drawn perpendicular to the longest diameter of erythema (the orthogonal diameter) and also measured. These two lengths were arithmetically summed, according to a standard, published protocol, as delineated in Figure 1[36]. The same procedure was used to quantitate the wheal response to histamine, which was, in all cases, smaller than the erythema measurement. The intra-individual coefficient of variation of this histamine skin prick testing is reported to be 20% [37]. In the current study the coefficient of variation in flare response to histamine was 32% in controls. Peak reactions, irrespective of anatomical location, and site-specific wheal and flare responses were compared between MAS patients and controls. Epinephrine pens were available at the bedside during skin testing.

### Genetic testing

The three study subjects who displayed anaphylactic reactions underwent genetic testing for mutations in GNAS in peripheral blood. Mutational analysis of the Arg 201 locus of GNAS was performed using site-specific polymerase chain reaction (PCR), restriction digestion and DNA sequencing.

### PBMC isolation

PBMCs were isolated from heparinized blood using Histopaque 1077 (Sigma-Aldrich, St. Louis, MO). Cells were pelleted, and resuspended in Buffer RLT Plus from the RNeasy^®^ Mini Plus Kit (QIAgen, Valencia, CA) and stored at -80°C until further processing.

### RNA isolation and quantitation

Samples were thawed, vortexed, and placed in QIAshredder columns and processed according to the manufacturer’s directions. RNA was examined by spectrophotometry and quantitated using the Ribogreen assay (Invitrogen, Carlsbad, CA). Ribogreen reagent was added to samples and standards, and samples were read on a Bio-Tek FL 800 fluorescent plate reader. RNA was diluted in nuclease-free water to a final concentration of 150 ng/ml cDNA. RNA quality was assessed by spectrophotometry and by the cycle number for the housekeeping gene, GAPDH.

### Reverse transcription and one-step real time PCR

DNA was digested using DNase 1, and residual DNase activity was quenched with EDTA. Reverse transcription was then performed using the SuperScript II Reverse Transcription Kit (Invitrogen, Carlsbad, CA) according to the manufacturer’s instructions. One-step real-time RT-PCR was performed using the SYBR Green PCR kit (Bio-Rad, Hercules, CA). G protein sequences were obtained from the Gene Bank database and the following primers were constructed Using the GenBank sequence for GNAS NM_ 000516: G_s_α sense: 5′-TCT ACC GGG CCA CGC ACC GC-3′; G_s_α antisense: 5′-GCA GGA TCC TCA TCT GCT TC-3′. We utilized the human GNAQ sequence NM_002072 to create the following primers: G_q_α sense: 5′-GAT GTT CGT GGA CCT GAA CC-3′; G_q_α antisense: 5′-CAA CTG GAC GAT GGT GTC CT-3′. BLAST searches were performed using the National Center for Biotechnology Information’s BLAST WWW Server. Commercially available primers were used for the housekeeping gene, GAPDH (Clontech, Mountain View, CA). The following parameters were used for the RT-PCR program for all genes: 95°C 3 min; 35 cycles of 95°C 20 sec, 56°C 20 sec, 72°C for 20 sec; 95°C 1 min; and 55°C 7 min. Data were calculated as the delta Ct ratio of the gene of interest compared to the delta Ct of the housekeeping gene (GAPDH). That ratio was also normalized as a percent of an internal standard control sample, prepared from a pool of PBMCs from healthy adult males and females.

### Protein quantitation

Protein concentrations were determined by the Bradford method utilizing a Coomassie protein assay kit from Pierce Biotechnology (Rockland, IL). A standard curve using bovine serum albumin was established from 0 to 2000 μg/ml. Sample (5 μl) was added to 96 well plates in duplicate. Coomassie reagent (250 μl) was added to each well and incubated for 10 minutes at RT. The absorbance was measured at 595 nm on a Power WaveX plate reader (Bio-Tek, Winooksi, VT).

### Immunoblot analysis for membrane-associated proteins

Total membrane protein (200 μg per well) was electrophoresed in 12% Tris–HCl gels and transferred to nitrocellulose membranes (Biorad, Hercules, CA) by electroblotting. Membranes were blocked in Tris-buffered saline with .1% Tween-20 (TBST) and 5% non fat dry milk, then incubated overnight with affinity-purified rabbit polyclonal IgG antibodies specific to each G protein (Santa Cruz Biotechnology, Santa Cruz, CA). The G_q_α antibody does not cross- react with the related G protein subunit, G_11_α. Membranes were incubated for 1 h in TBST with horseradish peroxidase conjugated 2° antibodies (Jackson Immunoresearch, West Grove, PA). Bands were visualized by chemiluminescence via western blot luminal reagent (Santa Cruz Biotechnology, Santa Cruz, CA) by exposure to Classic BX autoradiography film (MidSci, St. Louis, MO). The membranes were then stripped with western blot stripping buffer (Fisher Scientific, Hanover Park, IL) for 30 min at 37°C and re-probed for actin protein as an internal control, using a monoclonal primary antibody (MP Biomedicals, Santa Ana, CA). Bands were quantitated using densitometry and ImageQuant 5.2 software (Molecular Dynamics, Foster City, CA).

### Sequencing

G_s_α and G_q_α PCR fragments were treated with ExoSAP-IT (USB, Cleveland, OH) and subsequently sequenced using BigDye terminator v3.1 cycle sequencing chemistry and a 3730 capillary DNA analyzer (Applied Biosystems, Foster City, CA).

### Statistics

Differences in skin testing results between groups were assessed using 95% confidence intervals. G protein mRNA expression and IgE levels were compared by Wilcoxon rank sum tests. Because data were not normally distributed, Spearman correlation coefficients were determined comparing Tanner stage and peak histamine responses and between G protein mRNA and protein measurements.

## Results

### Atopic manifestations and hypersensitivities

Retrospective analysis revealed that ten out of 11 (90.9%) patients with MAS in this study displayed atopy and hypersensitivities encompassing a wide variety of reactions (Table 1). Allergic phenomena suggestive of H1 receptor activation included seasonal rhinitis, eczema, asthma, food allergies, drug reactions, latex allergies, and anaphylaxis. Activation of the H2 receptor was suggested by chronic gastritis, *H. pylori* negative ulcers, and gastroesophageal reflux noted in two patients. Type IV reactions, including severe bullous reactions to surgical tape occurred in two individuals. Anaphylaxis necessitating epinephrine administration had occurred in four of the 11 patients in the current study. Two of these patients experienced anaphylaxis after food ingestions, one after skin contact with latex, and one after receiving penicillin.

### Flare responses to histamine

**Figure 2:**
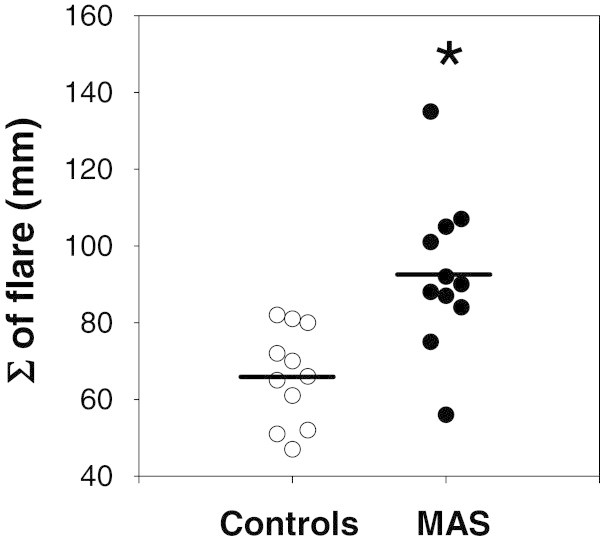
**Vertical dot plot depicting the sum of flare (Σ of flare) responses to histamine skin testing on the back in control participants and in patients with MAS.** * A significantly higher histamine response is seen in MAS patients compared to controls (n = 11 per group; p = 0.004).

Patients with MAS displayed significantly more vigorous flare responses to histamine when comparing the same anatomical site to that of controls. The sum of the skin flare response to histamine on the back was 92.7 ± 20.0 mm in MAS patients compared to 66.1 ± 12.4 mm in controls (mean ± S.D.) (95% confidence interval (CI) 11.8 to 41.4; p = 0.0013: Figure 2).

Similar flare responses were seen using the arm, although more variability was seen: 92.4 ± 30.2 mm in MAS patients compared to 68.3 ± 25.7 mm in controls (95% CI -0.8 to 49.0; p = 0.057).

The peak flare response to histamine irrespective of anatomical location was also significantly higher in patients with MAS compared to controls [104.4 ± 24.4 mm in MAS patients compared to 76.4 ± 12.9 mm in controls (95% CI 10.6 to 45.4; p = 0.003)].

### Wheal responses to histamine

**Figure 3:**
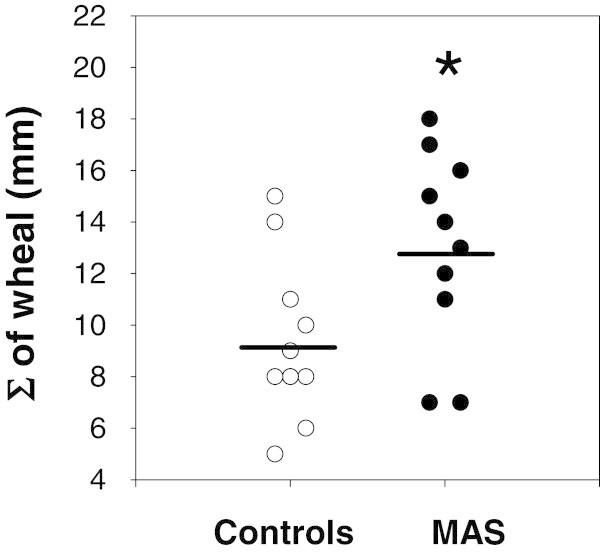
**Vertical dot plot depicting the sum of wheal (Σ of wheal) responses to histamine skin testing on the back in control participants and in patients with MAS.** Each group included one patient whose wheal measurement was not recorded. *A significantly higher histamine response is seen in MAS patients compared to controls (p = 0.01).

Patients with MAS also displayed significantly more vigorous wheal responses to histamine compared to controls in the back only. The sum of the mean skin wheal response to histamine on the back was 12.8 ± 3.7 mm in MAS patients compared to 9.3 ± 3.1 mm in controls (95% CI 0.46 to 6.54; p = 0.026; Figure 3).

No statistically significant differences in wheal responses in the arm were seen: 13.7 ± 3.8 mm in MAS patients compared to 13.1 ± 5.3 mm in controls (95% CI -3.50 to 4.70; p = 0.76). The differences in the peak wheal response to histamine irrespective of anatomical location between patients and controls also did not reach statistical significance 14.5 ± 4.1 mm in MAS patients compared to 13.6 ± 5.4 mm in controls (95% CI -3.364 to 5.164; p = 0.66).

### Responses to codeine

No differences were seen in wheal or flare responses to codeine. The sum of the skin flare response to codeine on the back was 37.8 ± 39.3 mm in MAS patients compared to 24.7 ± 26.3 mm in controls (95% CI -16.6 to 42.8;p = 0.36). The sum of the skin wheal response to codeine on the back was 8.8 ± 5.9 mm in MAS patients compared to 6.3 ± 2.7 mm in controls (95% CI -1.7 to 6.7; p = 0.22).

### G protein mRNA expression

**Figure 4:**
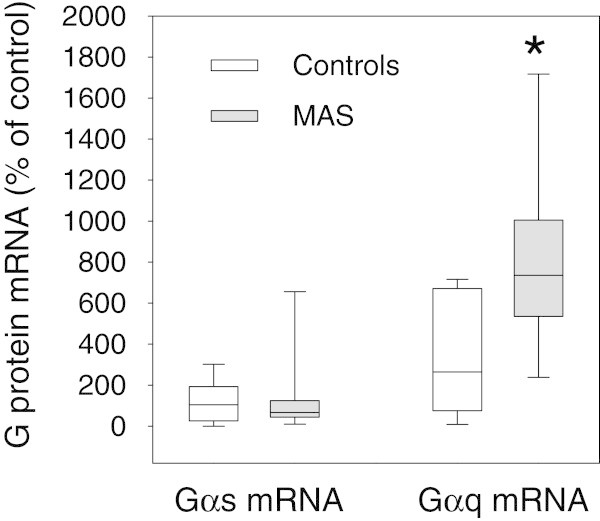
**Whisker plot of Gαs mRNA and Gαq mRNA in controls versus MAS participants.** Sufficient quantities and quality of mRNA were available on 9 to 11 participants per group. Although no differences are seen in Gαs mRNA, * Gαq mRNA is significantly higher in MAS patients compared to controls (p = 0.039). The solid lines within the boxes represent median values.

Sufficient quantities and quality of mRNA for PCR analysis were obtained from 9 to11 participants per group. No statistically significant differences between G_s_α mRNA levels were seen in patients versus controls. In contrast, MAS patients displayed significantly elevated median levels of G_q_α mRNA compared to controls (Figure 4; n = 9-11 per group; p =0.039). This difference was confirmed using the larger data which included two sex-matched, Tanner stage matched control participants for every patient with MAS. The median G_q_α mRNA level in controls was 386.38 versus 735.8 in MAS (p = 0.036; data not shown).

### G protein expression

Sufficient quantities of protein for immunoblot analysis were obtained from nine participants per group. No statistically significant differences between G_s_α or G_q_α protein levels were seen in patients versus controls. A direct positive correlation was seen between G_q_α mRNA and G_q_α proteins, but in control participants only (rho = 0.61; p = 0.03; data not shown).

### Effect of café-au-lait spots

No significant differences in wheal or flare responses to histamine, codeine, or saline were observed between the affected skin lesions to the contralateral unaffected skin site.

### Effect of severity of disease

A regression analysis was performed comparing the number of traditional manifestations of MAS and peak flare in response to histamine. No correlation was seen, Spearman (r) = - 0.22; p = 0.477.

### Effect of puberty

**Figure 5:**
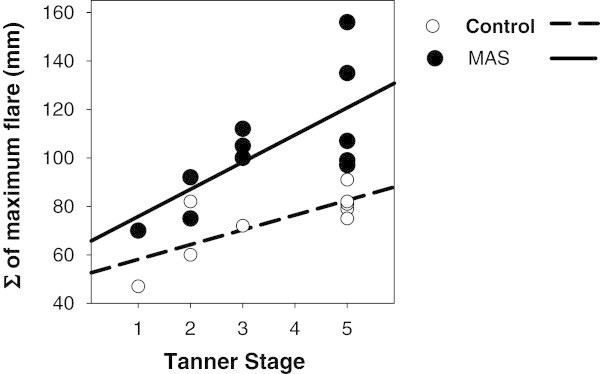
Regression analysis demonstrating a direct, positive correlation between Tanner stage and the maximum sum of flare responses to histamine skin testing irrespective of anatomical site in patients with MAS (n = 11; Spearman r = 0.67; p = 0.02) and in healthy control participants (n = 9; Spearman r =0.64; p = 0.03).

A regression analysis comparing Tanner stage to the maximum histamine flare response irrespective of anatomical site showed significant direct positive correlations in both patients with MAS and in control participants. The Spearman correlation coefficient (r) for MAS patients was 0.67; p = 0.02. The Spearman r for control participants was 0.64; p = 0.03; Figure 5.

### IgE levels

The mean IgE level in MAS patients was 653.1 ± 1195.9 kU/L and 81.0 ± 100.7 kU/L in controls. This difference did not reach statistical significance (p = 0.149).

### Genetic testing

An Arg 201 mutation was detected in exon 8 in one of the three participants who underwent genetic testing after experiencing anaphylaxis. This is patient #1, the most severely affected patient.

## Discussion

We have identified a high rate of allergic phenomena and hypersensitivities in patients with MAS compared to controls. In the current study, ninety-one percent of patients exhibited allergic phenomena ranging in severity from medication allergies to anaphylaxis. This is the first such report of atopy in McCune Albright syndrome in the medical literature. These observations ranged from classic IgE mediated phenomena to classic Type IV hypersensitivity reactions such as contact dermatitis. The wide range of sensitivities seen may result from the fact that numerous neurotransmitters, chemokines, cytokines, leukotrienes, and vasoactive peptides exert their actions via stimulatory G proteins, and predominantly via G_s_α.

We have also observed a heightened wheal and flare response to histamine skin testing in MAS patients. This observation may be considered surprising, as the molecular defect in MAS is a constitutive activation of G_s_α. Of all the histamine receptors, only H2 receptors signal via G_s_α. Although H2 receptors are important in gastric acid secretion, they are thought to play a minor role in allergy and in urticaria. In those conditions, H1 receptors, which signal via G_q_α, are thought to play predominant roles.

We did not observe a statistical increase in wheal and flare responsiveness to codeine in MAS patients compared to controls. If the heightened wheal and flare responses could be attributed solely to effects of histamine, one might have expected similar results with codeine as with histamine, as codeine induces histamine release by mast cells [34]. The differences in responsiveness to codeine and histamine could relate to the fact that codeine exerts its actions via an IgE independent mechanism and via classic opioid receptors, which signal via G_q_α as well as via inhibitory G proteins [35]. Codeine has been shown to exert additional actions in mast cells, including cytokine and chemokine release [35].

It is possible that the exaggerated histamine responses observed herein may have been amplified by gonadal hormones. The hypothalamic pituitary gonadal axis is known to be activated in female patients with MAS with sexual precocity. In support of this hypothesis, previous studies show a variation in histamine skin testing responses during the menstrual cycle and a positive correlation between serum estradiol and LH levels in females [29,30]. We did not measure gonadotropins of estradiol in our population, as these are dramatically altered by the stage of the menstrual cycle. Only one of our patients exhibited regular menstrual cycles. We used Tanner staging as a surrogate for chronic hormone exposure. Although our numbers are small, we have demonstrated statistically significant positive correlations between histamine responses and Tanner stage both in our MAS population and in healthy control participants.

We speculate that the aberrant activation of the hypothalamic pituitary gonadal axis in MAS may contribute to the activation of G_q_α and the H1 receptor pathways. In fact, we have previously demonstrated that exposure to estradiol or GnRH leads to a transcriptional upregulation of G_q/11_α [32]. Herein, we observed an upregulation in G_q_α mRNA expression in MAS participants compared to controls and confirmed these finding in a larger sample size. It is perhaps not surprising that we did not observe a transcriptional upregulation in G_s_α in MAS patients. This may relate to the mosaicism in this condition. In the presence of an activating mutation of the GNAS gene in MAS, further transcriptional upregulation of GNAS would not seem to be adaptive.

The constitutive activation of a stimulatory G protein in the clinical condition of MAS may offer important insights into mechanisms in histamine signaling. Constitutive activity of one GPCR in the absence of ligand binding may have important implications for histamine “cross talk.” It is interesting to note that antihistamines are actually inverse agonists, meaning that they can reduce histamine receptor constitutive activity in the absence of binding of any histaminic ligand. Our results support the possibility that the constitutive activation of one G protein coupled histamine receptor, namely G_s_α, may offer a permissive effect on G_q_α activity. Numerous in vitro studies examining other agonists that signal through GPCRs have shown that expression of constitutively active stimulatory G protein coupled receptors allows for enhanced signaling of other co-expressed G protein receptors [23-25]. In fact, synergism has been demonstrated between H1 and H2 receptors, with cross talk between the G_q_α/PKC and the G_s_α/PKA pathways [13,20,27].

One proposed mechanism for these observations is that both H1 and H2 agonists compete for clearance by the same cytochrome P450 isoenzyme [38]. This mechanism could not explain the numerous reports of in vitro cross talk between H1 and H2 receptors, however [13,27]. Again, our data are consistent with the concept that the constitutive activation of G_s_α may offer a permissive effect on G_q_α -coupled receptors.

Human clinical studies also support the concept of “cross talk” between H1 and H2 action in dermatologic manifestations: H2 blockers have been noted to be clinically beneficial in the management of chronic urticaria and dermographism [26,39,40]. A recent meta-analysis showed that a combination of ranitidine with diphenhydramine was slightly more effective at improving the resolution of urticaria than diphenhydramine administered alone [41]. However, the authors concluded that the review did not allow confident decision-making about the use of H2 receptor antagonists for urticaria [41].

A limitation of the study was that it is difficult to positively confirm the diagnosis in MAS. Genetic testing by PCR methods currently is available, but it detects the mutation in peripheral blood in only an estimated 40% of individuals because of the mosaicism of this condition. The detection rate can be increased significantly when cellular bony tissue is available. The diagnosis of McCune Albright syndrome remains a clinical one, which necessitates the presence of at least two features of the classic triad [11].

Another limitation of the study was that the study numbers are small. If increased atopy or excessive histamine responsiveness can be confirmed in a larger group of patients, especially in patients with molecularly confirmed MAS, inclusion of allergic phenomena among the supporting diagnostic criteria of MAS may be warranted. Additional supportive diagnostic criteria would be extremely clinically useful in the management of patients with MAS. A high frequency of *formes frustes* variants exists in this condition. For example, patients with isolated monostotic and polyostotic fibrous dysplasia have been shown to display activating mutations in GNAS without the full spectrum of McCune Albright syndrome [42-44]. Variable tissue expression, mosaicism, and non-confirmatory genetic testing in this condition all lead to underdiagnosis of MAS.

Another limitation of the study was the lack of a dose–response curve. If MAS patients indeed possess exaggerated wheal and flare responses to both histamine and to saline compared to controls, as our data suggest, perhaps the differences between MAS patients and controls could have been further amplified at much lower doses of histamine.

The current study raises the possibility that in vivo or in vitro testing for heightened responsiveness to *other* G protein-linked endocrine or non-endocrine mediators could eventually lead to improved clinical diagnostic testing for MAS.

The current findings, which have implications in both the diagnosis and management of MAS, indicate a need for confirmatory studies in a larger series of patients with genetically confirmed MAS.

## Competing interests

There is no conflict of interest that could be perceived as prejudicing the impartiality of the research reported.

## Authors’ contributions

JDJ and ALT collected and recorded data. SAS performed most of the molecular studies. JDJ and SAS analyzed data. JDJ, SAS, and ALT wrote the manuscript. All authors read and approved the final manuscript.
